# Therapeutics for Chemotherapy-Induced Peripheral Neuropathy: Approaches with Natural Compounds from Traditional Eastern Medicine

**DOI:** 10.3390/pharmaceutics14071407

**Published:** 2022-07-05

**Authors:** Geehoon Chung, Sun Kwang Kim

**Affiliations:** 1 Department of Physiology, College of Korean Medicine, Kyung Hee University, Seoul 02447, Korea; geehoon.chung@khu.ac.kr; 2 Department of Korean Medicine, Graduate School, Kyung Hee University, Seoul 02447, Korea

**Keywords:** chemotherapy-induced peripheral neuropathy, traditional eastern medicine, neuropathic pain

## Abstract

Chemotherapy-induced peripheral neuropathy (CIPN) often develops in patients with cancer treated with commonly used anti-cancer drugs. The symptoms of CIPN can occur acutely during chemotherapy or emerge after cessation, and often accompany long-lasting intractable pain. This adverse side effect not only affects the quality of life but also limits the use of chemotherapy, leading to a reduction in the survival rate of patients with cancer. Currently, effective treatments for CIPN are limited, and various interventions are being applied by clinicians and patients because of the unmet clinical need. Potential approaches to ameliorate CIPN include traditional Eastern medicine-based methods. Medicinal substances from traditional Eastern medicine have well-established analgesic effects and are generally safe. Furthermore, many substances can also improve other comorbid symptoms in patients. This article aims to provide information regarding traditional Eastern medicine-based plant extracts and natural compounds for CIPN. In this regard, we briefly summarized the development, mechanisms, and changes in the nervous system related to CIPN, and reviewed the substances of traditional Eastern medicine that have been exploited to treat CIPN in preclinical and clinical settings.

## 1. Introduction

Chemotherapy-induced peripheral neuropathy (CIPN) is a major adverse effect that may limit the use of cancer chemotherapy agents [1,2]. CIPN usually manifests as sensory neuropathy symptoms in patients. This involves the alteration of sensation induced by mechanical and/or thermal stimuli. Various molecular mechanisms and cellular alterations related to CIPN have been revealed in recent decades. Chemotherapeutic agents act at multiple levels of the somatosensory nervous system, thus affecting the expression of ion channels, myelination of the nerve fibers, the excitability of the sensory neurons located in the dorsal root ganglion and spinal cord, and the connectivity of the pain-related brain network. These changes eventually distort the transmission of sensory signals, causing the normal peripheral sensation to become painful. Despite numerous efforts, there is no effective clinical method for the treatment of CIPN [3,4,5]. Several patients with cancer stop chemotherapy due to this adverse effect. Pain from CIPN not only impairs the quality of life but also becomes a life-threatening factor in these patients by limiting the available cancer treatments [2,3].

As indicated by its name (“peripheral” neuropathy), it is evident that peripheral mechanisms are the initial causes of the development of these symptoms. Recent review papers have described the multiple mechanisms that induce sensory symptoms in CIPN [2,6]. However, the mechanisms of the maintenance of CIPN symptoms are not limited to peripheral changes. Although the initial cause of CIPN is in the peripheral nerve, prolonged injury signals cause abnormalities in the higher levels [7]. This leads to chronic pain, such as neuropathic pain caused by other reasons [8,9]. Previous studies in the research field of general neuropathic pain revealed that central changes, as well as peripheral changes, are critically involved in chronic pain states. Damage to the peripheral nerve that causes pain could induce alterations at multiple levels of the somatosensory nervous system. This includes the spinal cord and brain. These changes could amplify pain transmission even after the initial damage is recovered [10]. Similar to the chronic neuropathic pain caused by other reasons, CIPN symptoms are often prolonged after cessation of the chemotherapeutic agent, and in some cases, the symptoms persist [1,11,12].

Various therapeutic methods have been used to attenuate the symptoms of CIPN in patients. The treatment options include medications from traditional Eastern medicine, due to their beneficial effects in relieving numerous symptoms of CIPN [13,14,15]. Generally, in traditional Eastern medicine, it is recommended to choose multiple substances from many options and combine them for a therapeutic effect against the target symptoms. Treatment strategies should be selected according to individual differences in patients’ general conditions and patterns of comorbid symptoms when applying the traditional Eastern medicine approach. The current understanding of the mechanisms shows that the analgesic effects of traditional Eastern medicinal substances against CIPN symptoms can be induced at different levels of the nervous system. An adequate combination of treatment methods that act on multiple targets or work synergistically would help develop new treatment options for CIPN. In addition, the effects of many substances on cancer have been studied extensively in recent decades [16,17,18,19,20]. Considering that the treatment for CIPN should not deteriorate cancer, these pre-established results are advantageous for selecting the traditional Eastern medicine approach as a treatment option.

**Figure 1:**
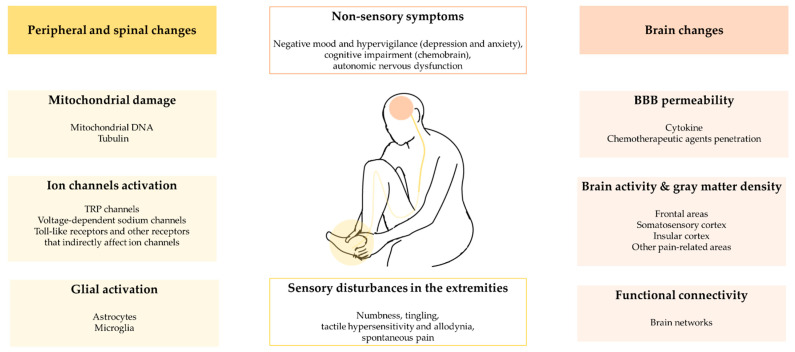
The summary of peripheral, spinal and brain changes in CIPN.

In this article, we first review the causes of CIPN and its symptoms for a general understanding of CIPN (Figure 1). The peripheral and spinal mechanisms studied in patients or animal models, and brain changes associated with prolonged pain are also summarized. We further discuss the recent studies seeking novel interventions for CIPN. Particularly, we focus on the methods using plant extracts and natural compounds from traditional Eastern medicine. Considering the clinical difficulties in the investigation of new drugs, utilizing traditional medical knowledge is advantageous for screening drug candidates with long-term safety and effectiveness. In this respect, recent studies on phytochemicals, extracts from medicinal plants, herbal decoctions, and animal venom substances used in traditional Eastern medicine have been summarized and discussed.

## 2. Development of CIPN Symptoms by Chemotherapeutic Agents

Chemotherapeutic agents cause damage to the nervous system, leading to the induction of CIPN. In particular, patients treated with the platinum-based chemotherapeutic agent oxaliplatin or the taxane-based agent paclitaxel are predisposed to develop immediate peripheral neuropathy. Other chemotherapeutic agents, including vinca alkaloids, proteasome inhibitors, and immunomodulatory drugs, also induce CIPN symptoms [6,21]. Sensory symptoms are the most prominent side effects of chemotherapeutic agents. However, chemotherapeutic agents also induce side effects such as dizziness, cognitive dysfunction, and motor dysfunction. Approximately 80% of the patients treated with these agents develop CIPN within 6 months. Pain symptoms are the major cause that limits their use. Most patients reported pain within 6.5 months of the use of the chemotherapeutic agent [11]. Even if the patient does not show pain symptoms in the early period of chemotherapy treatment, the stochastic rate of pain development increases with the duration of chemotherapeutic drug use. Approximately one-third of patients have chronic CIPN six months or more after the end of chemotherapy [22]. In a subset of the patients, the symptoms of CIPN deteriorate after the cessation of chemotherapeutic agents [2,23]. This “coasting” phenomenon includes worsening of the preexisting mild CIPN or development of new CIPN. In this regard, the management of CIPN has to be continued after the cessation of chemotherapy. More so, the sensory symptoms of the patients have to be observed throughout the period.

Although the types and severity of CIPN symptoms can vary depending on the chemotherapeutic agent used, many patients report sensory disturbances in the extremities. Numbness, tingling, mechanical/thermal hypersensitivity, and spontaneous pain are common symptoms. The representative pain symptoms include cold allodynia, which was observed in most patients treated with oxaliplatin or paclitaxel. Heat hyperalgesia is relatively rare, and several patients have reported a loss of heat sensitivity with these agents [24]. Pain-related distress is accompanied by depression, anxiety, and other symptoms related to hypervigilance and negative mood. Mental distress and physical pain influence patients to cease chemotherapy.

Similar to other neuropathic pain caused by nerve damage, the discomfort of pain symptoms caused by chemotherapeutic agents is not controlled by conventional analgesic drugs. Patients rarely adapt to the pain symptoms. Rather, the intensity of pain and discomfort in patients with CIPN often increase with the continued use of chemotherapeutic agents. There have been attempts to suppress CIPN symptoms with treatments such as pregabalin, venlafaxine, or duloxetine. However, only a subset of patients reported having relief from symptoms, which was restricted to partial relief [25].

## 3. Peripheral and Spinal Mechanisms of the CIPN

Platinum-based chemotherapeutic agents, such as oxaliplatin and cisplatin, induce oxidative stress in the mitochondria. These agent molecules act on mitochondrial DNA in cells and impair the respiratory chain in mitochondria, promoting the production of reactive oxygen species [26,27]. Thus, the apoptotic pathway is activated in cancer cells. Hence, these chemotherapeutic agents easily exert an anti-cancer effect. However, the target cells of these drugs are not restricted to cancer cells. These mechanisms may also affect non-cancer cells, resulting in adverse effects, such as CIPN. Damage to the primary afferent nerve fibers is claimed to be the primary cause of the abnormal pain symptoms in CIPN. The primary afferent fibers of the sensory nerve relay information from the cutaneous skin to the spinal cord, and have very long axons. Chemotherapeutic agents easily affect the mitochondria within sensory afferents due to this characteristic. In the case of paclitaxel, a taxane-based chemotherapeutic agent, alteration of mitochondrial function was claimed to be responsible for the manifestation of CIPN [28]. Many other chemotherapeutic agents also induce mitochondrial dysfunction by altering microtubule dynamics [29,30,31,32]. This effect is exerted by the interaction between the drug and tubulin. In addition to its effect on microtubule dynamics, tubulin can also alter mitochondrial function via its direct interaction with voltage-dependent anion channels located in the mitochondrial membrane [30]. Changes in tubulin have been reported to be common downstream in taxane-based drugs, vinca alkaloids, and proteasome inhibitors [31].

Chemotherapeutic agents also induce abnormal sensory signals and amplify pain through their action on ion channels in the cellular membrane [21,33,34]. These phenomena are mediated by activation of TRP (transient receptor potential) channels [33,35], voltage-dependent sodium channels [34], toll-like receptors [36], and/or other various receptors that indirectly affect ion channels [35,37,38]. The activation of ion channels in the primary afferent fibers also evokes nerve signals similar to the activation of nociceptors in the periphery endings. Accordingly, sensory neurons in the dorsal root ganglion (DRG) fire action potentials that are then transmitted to various brain regions via the spinal cord; thus, the individual perceives the sensory signals as painful distress.

Another mechanism involves glial activation [21,39,40]. For example, the myelin sheath can be damaged by chemotherapeutic agents. The peripheral nerve fibers are myelinated by Schwann cells, and damage or inflammatory changes in this glial cell can amplify the signal transmission of sensory nerve fibers. Glial mechanisms also include changes in the microglia and astrocytes in the central nervous system [41]. Interestingly, the recruitment of microglia and astrocyte in the development of CIPN appears to be related to the chemotherapeutic agent used. Generally, the activation of astrocytes plays a key role in the pathogenesis of CIPN. For example, oxaliplatin affects astrocytes in the spinal cord by activating adenosine kinase and subsequent reduction in adenosine signaling. Activation of astrocytes alters the signaling of other glial cells and neighboring neurons, facilitating signal transmission in the spinal cord [42]. Astrocyte activation was also observed in paclitaxel- and bortezomib-induced CIPN [43,44]. In contrast to the general recruitment of astrocytes across multiple CIPN models, the role of microglial activation in CIPN is somewhat vague and seems to be specific to the chemotherapeutic agent used. In the CIPN model induced by oxaliplatin, paclitaxel, or bortezomib, microglia were not activated in the spinal cord and did not appear to play a significant role in CIPN [43,44]. Conversely, activation of microglia, but not astrocytes, was persistently observed in the spinal cord in the CIPN model induced by cisplatin [45].

All the peripheral and spinal mechanisms are important for the development and maintenance of CIPN. Attempts have been made to develop novel intervention methods based on these mechanisms. Some have shown significant results in clinical trials. However, no treatment has sufficiently suppressed pain symptoms in human patients. Little preclinical understanding of the CIPN is being translated into clinical trials. As such, substances that were found to be promising in the preclinical setting have failed in clinical trials [46].

## 4. Brain Changes Observed in Subjects with CIPN

The direct effects of chemotherapeutic agents on the brain are unclear in many aspects. Drugs that cause CIPN, such as cisplatin, oxaliplatin, or paclitaxel, are thought to affect the peripheral nervous system rather than the brain. This is because these drugs do not pass through the blood–brain barrier (BBB) [47]. However, studies have shown that small amounts of these substances are found in the brain when they are injected systemically. For example, early studies reported that chemotherapeutic agents, such as cisplatin or doxorubicin, could exert anti-cancer effects against brain tumors, despite limited passage across the BBB [48,49]. Meanwhile, a study reported that a radiotracer form of paclitaxel was found in the brain of animal models when the tracer was intravenously injected, although the amount of the tracer measured in the brain was very small [50]. This shows that the BBB does not completely prevent the penetration of these drugs into the brain. A recent review discussed that cisplatin could migrate into the brain, despite its limited passage across the BBB through the copper transporter 1-mediated mechanisms [51]. Other studies have shown that oxaliplatin affects epithelial cells that constitute the BBB, thereby increasing the chances of penetration of oxaliplatin and cytokines into the brain [52]. This invokes the local release of cytokines in the brain and enables the entrance of more chemotherapeutic agents and cytokines [53,54]. Chemobrain, another major complaint of patients treated with chemotherapy, might be related to this mechanism. Several patients treated with chemotherapy experience cognitive impairment, autonomic nervous dysfunction, decreased motor skills, and other general neurological symptoms. These symptoms may also occur due to the direct effect of chemotherapeutic agents on the brain and/or indirect effects on the BBB [51,53,54].

A few studies investigated the brain-level alterations accompanied by chronic pain symptoms in patients with CIPN [7]. Elaine et al. analyzed the brains of patients with CIPN using fMRI and reported that nociception-related brain regions undergo plastic changes due to pain symptoms [55]. Nudelman et al. analyzed cerebral perfusion and gray matter density of patients with CIPN using MRI [56]. They found that the patients showed increased cerebral perfusion in the frontal areas. They also reported that the degree of CIPN symptoms and related perfusion changes were correlated with the change in gray matter density. Nagasaka et al. studied altered brain activity using fMRI in a macaque model of CIPN [57]. The model animals injected with oxaliplatin showed increased activation in the somatosensory and insular cortices in response to cold stimulation. This was attenuated by duloxetine, a selective serotonin and norepinephrine inhibitor. In addition, inactivation of the brain regions with focal microinjection of GABA agonist muscimol ameliorated cold hypersensitivity symptoms. This indicates that the brain change was in part responsible for the expression of the CIPN. Another recent fMRI study performed by Yeh et al. reported a change in brain connectivity in patients with CIPN [58]. They analyzed brain networks as 11 different subtypes and found that patients treated with analgesic treatment (auricular point acupressure) reported reduced pain and their brain connectivity pattern shifted to a different subtype of the brain network. The difference between the brains of patients with CIPN and the control group did not tell the causal relationship in many reports; however, studies have shown that CIPN symptoms could be ameliorated by various methods related to the manipulation of brain activity [59,60,61].

## 5. Candidates for New Therapeutics: Approach with Knowledge from Traditional Eastern Medicine

**Figure 2:**
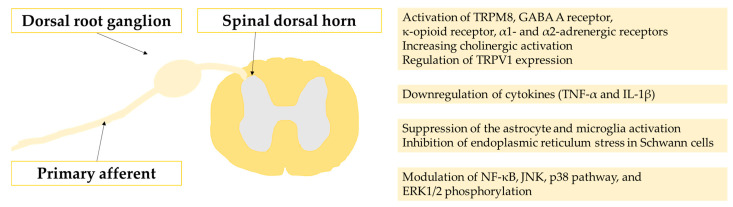
The molecular actions of the substances presented in the paper.

Despite efforts to seek therapeutics for CIPN, heterogeneity in the prevalence, time to symptom incidence, and preexisting cancer condition of the patients make drug development difficult. The ideal intervention for CIPN should not interfere with the general condition of the patients with cancer, or the efficacy of the anti-cancer effect of the chemotherapeutic agents [62,63]. Substances that deteriorate the progression of cancer should be avoided even if they can suppress the pain of patients. One promising approach is to use knowledge from traditional Eastern medicine. Many substances used in traditional Eastern medicine have been studied for decades, and multiple studies have investigated their effects on cancer [16,17,18,19,20]. Furthermore, traditional Eastern medicine has a distinctive classification method for the effects of therapeutic substances. For example, some medicinal plants and natural chemicals are known to relieve “cold” symptoms of the body, including cold stimuli-related pain [64,65]. Considering that the representative symptoms of CIPN in patients treated with oxaliplatin or paclitaxel are cold-evoked allodynia, these “cold-relieving” medicinal substances from Eastern medicine could be promising candidates for new therapeutics for the symptoms [66,67,68]. In the following subsections, the effects of traditional medicinal substances on CIPN symptoms are reviewed. The preclinical and clinical studies of each substance are also summarized in Table 1. The molecular actions of the substances are briefly summarized in Figure 2.

### 5.1. Aconitum

In traditional Eastern medicine, substances derived from Aconitum have been used to warm meridian channels, thus ameliorating pain and numbness. Consistent with the traditional knowledge, Suzuki et al. found that Aconiti radix, the root of Aconitum carmichaeli, could alleviate oxaliplatin-induced mechanical and cold pain symptoms in mice with CIPN. This analgesic effect was mediated by its constituent neoline [69]. Jung et al. showed that Aconiti tuber could alleviate neuropathic pain symptoms in rats treated with oxaliplatin by suppressing the activation of astrocytes in the spinal dorsal horn and downregulating the production of pro-inflammatory cytokines, including TNF-α and IL-1β [65]. Previous studies found that bulleyaconitine A, an alkaloid isolated from Aconitum plants, could alleviate paclitaxel-induced CIPN symptoms by inhibiting synaptic transmission in C-fibers and the spinal cord [70,130]. In a clinical retrospective case series study, Aconiti radix-containing herbal formulas were shown to be beneficial for human patients with CIPN symptoms [131]. Although the effects were proven in the clinical and preclinical settings as shown above, Aconitum should be carefully administered to patients because it is toxic and can be lethal if not processed properly [132,133].

### 5.2. Astragalus

Astragalus belongs to the Fabaceae family and is believed to increase overall vitality. Astragali radix, the dried root of Astragalus membranaceus, has long been used as a remedy for various chronic diseases associated with energy shortages caused by an overactive immune system. Previous studies have shown its pharmacological action in modulating the immune system and its beneficial effects as an adjunct cancer therapy. Studies showed that Astragali radix extracts reduced oxaliplatin-induced pain symptoms via suppression of microglia and astrocyte activation [72,73]. Notably, the treatment showed a neuroprotective effect not only in the peripheral nerves and spinal cord, but also at the level of the brain [73]. Human studies also showed that the administration of this herb is beneficial for the prevention and treatment of neurotoxicity induced by oxaliplatin [134,135]. Deng et al. analyzed the effect of an Astragali radix-based herbal prescription (Wen-Luo-Tong) on CIPN symptoms in human patients and concluded that it is clinically effective for relieving symptoms [136,137]. Lee et al. showed the other Astragalus-based herbal prescription (SH003) could alleviate docetaxel-induced neuropathic pain [138]. Studies also showed that the components included in Astragali radix have an anti-cancer effect [139,140] and help to prevent nephrotoxicity and hepatotoxicity induced by the anti-cancer drugs [73]. These further suggest the usefulness of this substance in anti-cancer therapy.

### 5.3. Coptis

Coptis chinensis and Coptis teeta have been medicinally used in Eastern Asian regions for treating symptoms related to “damp-heat” of the body, such as fever, swelling, or dyspepsia. Berberine, a monomeric alkaloid compound included in Coptis, has been studied in previous studies and proven to exert anti-cancer effects by multiple mechanisms [141,142,143,144,145]. A recent study showed that berberine could alleviate CIPN symptoms induced by cisplatin treatment [74] as well. The authors of the study found that the alleviating effect of berberine on CIPN was mediated by the regulation of TRPV1 expression in DRG neurons. NF-κB expression and JNK/p38 MAPK/ERK pathways were involved in this modulatory effect of berberine.

### 5.4. Cinnamommum

Cinnamomum cassia Presl, a cinnamon tree (broiler tree) of the camphor family, has been widely used in East Asia to treat the symptoms of various diseases. Cinnamomi cortex is a representative “warm” medicinal herb and is believed to improve blood circulation, enhance the immune system, alleviate inflammation, and exert an analgesic effect. Studies have found that Cinnamomi cortex extract could attenuate oxaliplatin-induced mechanical and cold hypersensitivity in a rodent model of CIPN [66,67]. Kim et al. found that treatment with Cinnamomi cortex reduced CIPN symptoms by the suppression of glial activation and inhibition of IL-1β and TNF in the spinal cord [66]. The authors also revealed that coumarin and cinnamic acid in the Cinnamomi cortex are the major compounds responsible for the analgesic effect. Both these compounds also have anti-cancer effects [146,147,148,149].

### 5.5. Curcuma

Curcumin, a chemical from Curcuma longa plants, is known to promote blood circulation, eliminate blood stagnation and ameliorate pain. The analgesic effect of curcumin on CIPN symptoms can be attributed to multiple actions [75,76,77,150]. Zhang et al. suggested that curcumin alleviates oxaliplatin-induced pain symptoms by inhibiting the oxidative stress-mediated activation of NF-κB and mitigating inflammation [77]. Babu et al. suggested that the analgesic effect of curcumin on vincristine-induced pain is mediated by the suppression of pro-inflammatory cytokines [75]. Curcumin also has potent anti-cancer effects [151]. In addition, the co-administration of curcumin with chemotherapeutic agents induces synergistic action to increase anti-cancer activity [152,153,154,155]. Studies have shown that any drug administered in combination with curcumin could improve the anti-cancer effect [155,156,157,158,159].

### 5.6. Dryobalanops

Borneol, an organic terpene derivative, has been isolated from several plant species. In traditional medicine, natural borneol derived from Dryobalanops aromatica has been used to restore consciousness, remove heat, and relieve pain. It has been shown to be safe for human and is currently approved by the US FDA for use as a flavoring substance or adjuvant for food. Zhou et al. confirmed that borneol administration attenuated oxaliplatin-induced neuropathy in mice. The authors found that borneol exerted an analgesic effect against mechanical and cold allodynia via the blockade of transient receptor potential ankyrin 1 (TRPA1) [78]. Other studies found that the analgesic effect of borneol on general neuropathic pain also involves the activation of TRPM8 and GABA A receptors [160,161]. Notably, borneol transiently alters the permeability of the BBB, helping in drug delivery to the brain [162,163,164,165,166]. Multiple studies have shown that borneol treatment with chemotherapeutic agents could enhance the accumulation of the chemotherapeutic drug in the brain tissue and intracellular uptake, thereby improving the survival of glioma-bearing animals [167,168,169,170]. Borneol co-administered with temozolomide also enhanced anti-cancer efficacy of the drug against glioma by triggering mitochondrial dysfunction and ROS-induced DNA damage [171].

### 5.7. Lithospermum

Lithospermum belongs to the Boraginaceae family, and Lithospermi radix (the dried root of Lithospermum erythrorhizon) has been used to facilitate wound-healing and treatment of various inflammatory symptoms. Cho et al. showed that an aqueous extract of Lithospermi radix could ameliorate oxaliplatin-induced neurotoxicity, and suggested anti-inflammatory activities in the neuronal immune cells as the mechanisms for this effect [79]. The authors showed that treatment with Lithospermum suppressed the spinal activation of astrocytes and microglia, leading to the alleviation of mechanical hypersensitivity in oxaliplatin-induced neuropathy. The treatment also affected intraepidermal nerve fibers in the skin and DRG neurons. Yu et al. showed that herbal extract decoction containing Lithospermum could attenuate capecitabine-associated hand-foot syndrome in human patients. Shikonin, one of the main active ingredients of Lithospermum, has anti-nociceptive and anti-cancer effects [172,173,174], making it an attractive target for further research [175,176].

### 5.8. Paeonia

Paeonia lactiflora has been used to treat cardiovascular symptoms, muscle pain, and inflammatory and autoimmune diseases in traditional Eastern medicine. Paeoniae radix is included in various herbal formulas used to treat CIPN [68,177]. Paeoniflorin is a compound isolated from Paeonia lactiflora and has anti-oxidative, anti-inflammatory, and anti-cancer effects [178,179,180,181]. Studies have shown that paeoniflorin could alleviate nerve injury-induced neuropathic pain symptoms and suggested the suppression of the p38 MAPK pathway and NF-κB as the underlying mechanisms [182,183]. Andoh et al. showed that topical application of paeoniflorin prevented paclitaxel-induced mechanical allodynia by protecting sensory nerves from demyelination. This effect was mediated by the activation of the adenosine A1 receptor [80].

### 5.9. Plantago

Plantaginis semen, the dried seeds of Plantago asiatica, Plantago depressa, or Plantago major, have been used in traditional medicine to relieve heat symptoms. In addition, Plantaginis semen is one of the constituents of the Ucha Shinki Hwan (Gosha-jinki-Gan), an extensively studied herbal formula as a treatment for CIPN symptoms [81]. Andoh et al. suggested that the aqueous extract of Plantaginis semen ameliorated CIPN symptoms in paclitaxel-treated mice via its antioxidant activity [82]. The authors also found that aucubin and pedicularis-lactone, the major constituents of Plantaginis semen, played an important role in the anti-allodynic action [81,82,83,84]. The analgesic effect of aucubin on paclitaxel-induced mechanical allodynia is mediated by the inhibition of endoplasmic reticulum stress in peripheral Schwann cells [83]. The authors further studied pedicularis-lactone isolated from Viticis fructus, the dried fruit of Vitex rotundifolia Linné filius, instead of Plantaginis semen, and confirmed that this substance also showed a potent anti-allodynic effect. Unlike aucubin, the effect of pedicularis-lactone was not mediated by the inhibition of endoplasmic reticulum stress [84], and the underlying mechanisms are still unclear.

### 5.10. Sophora

Matrine is an alkaloid isolated from the herb Sophora flavescens, Sophora angustifolia, and Echinosophora koreensis. In traditional Eastern medicine, these herbs have been used as anti-inflammatory and analgesic drugs. The pain-relieving effect of matrine is mediated by multiple mechanisms. Studies showed that matrine exerted analgesic effects on vincristine-induced pain symptoms via its anti-oxidative, anti-inflammatory, and calcium antagonistic actions [85,86]. Other antinociceptive mechanisms of matrine include κ-opioid receptor agonism [184] and cholinergic activation in the nervous system [185]. Recent studies have shown that this substance can be used to reduce the side effects of opioid drugs, such as tolerance and withdrawal symptoms [186,187,188]. Matrine also has an anti-cancer effect, and could strengthen the anti-cancer capacity of other chemotherapeutic drugs [189,190]. Other beneficial effects of matrine include reversing anti-cancer drug resistance and reducing the toxicity of anti-cancer drugs [191].

### 5.11. Bee Venom Therapy

Bee venom has been traditionally used as a part of acupuncture therapy to treat diseases such as neuralgia, arthritis, facial paralysis, numbness, and various pain symptoms. Bee venom acupuncture, also referred to as apipuncture, involves the stimulation of acupoints with diluted bee venom. The main ingredients include melittin, apamin, and phospholipase A2. It has been used to exert healing effects in a variety of painful conditions in traditional Eastern medicine. CIPN can be mitigated or prevented by treatment with bee venom [87,88,90,91,92,93,95,97,98,99,192] or its constituents [89,94,193,194] in preclinical and clinical settings. Recent preclinical studies showed that the therapeutic effect is mediated by the activation of spinal α1- and α2-adrenergic receptors [92,94,193], although other mechanisms also exist [90,99,194]. Li et al. further suggested that the activation of spinal adrenergic receptors is mediated by the bee venom-induced release of norepinephrine from the locus coeruleus in the brain [96]. Bee venom treatment showed long-lasting and additive analgesic effects when combined with venlafaxine, a selective serotonin and noradrenaline reuptake inhibitor [90].

### 5.12. Pharmacopuncture Therapies

In clinical settings, pharmacopuncture therapy (acupoint injection of medicinal substances) is used to ameliorate the side effects of chemotherapy agents. A case report suggested that pharmacopuncture using the dried resin of Toxicodendron vernicifluum (Rhus verniciflua stokes) mixed with Cinnamomi cortex extracts ameliorated CIPN symptoms [102]. Another case study reported that snake venom pharmacopuncture could alleviate CIPN symptoms [100]. Yoon et al. showed that pharmacopuncture with Scolopendra subspinipes could suppress oxaliplatin-induced mechanical allodynia in mice, and the analgesic effect is mediated by the activation of α2-adrenergic receptors in the spinal cord [101].

### 5.13. Gyeji Ga Chul Bu Tang

Gyeji ga Chul Bu Tang (in Korean), also called Gui Zhi Jia Shu Fu Tang (in Chinese) and Keishi-ka-jutsu-bu-To (in Japanese), is a herbal formula including Cinnamomi cortex, Aconiti tuber, Atractylodis lanceae rhizome, Glycyrrhizae radix, Paeoniae radix, Zingiberis rhizoma, and Zizyphi fructus. This formula has been widely used for treating cold-related symptoms and pain symptoms in traditional medicine. Preclinical and clinical studies have shown that treatment with this formula has beneficial effects on CIPN symptoms. Cinnamomi cortex and Aconiti tuber are mainly responsible for relieving sensory symptoms [64,103,195].

### 5.14. Siwei Jianbu Tang

Siwei Jianbu Tang, a medicine created recently in China based on traditional medicinal knowledge, is made up of the following four herbs: Paeonia veitchii Lynch, Salvia miltiorrhiza Bge, Achyranthes bidentata Blume, and Dendrobium nobile Lindl. The main effect of this formula is to improve blood circulation, restore lower limb function, and relieve pain. Zhang et al. and Suo et al. showed that Siwei Jianbu could prevent CIPN symptoms by inhibiting the expression of NF-κB via the downregulation of phosphorylated ERK1/2 and p38 [104,105].

### 5.15. Ucha Shinki Hwan, Also Referred to as Jeseng Singi Hwan

Ucha Shinki Hwan (in Korean), also called Niu Che Shen Qi Wan (in Chinese), Gosha-jinki-Gan (in Japanese), Jeseng Singi Hwan (in Korean), and Ji Sheng Shen Qi Wan (in Chinese), is formulated from the following herbal ingredients: Cinnamomi cortex, Aconiti tuber, Rehmanniae radix, Achyranthis radix, Corni fructus, Moutan cortex, Alismatis rhizome, Dioscoreae rhizome, Plantaginis semen, and Poria sclerotium. In traditional medicine, this prescription has been used to treat numbness or cold hypersensitivity in the extremities, fatigue, and pain. Studies have found that treatment with this formula could ameliorate CIPN in preclinical [71,106,107,108,109,110,111,112,118] and clinical settings [113,114,115,196,197,198], with controversial prevention effects [116,117,196,197,199].

### 5.16. Yukgunja Tang

Yukgunja Tang, also referred to as Liu Jun Zi Tang (in Chinese), and Rikkunshi-To (in Japanese), consists of ginseng, Atractylodes, Poria cocos, Glycyrrhizae, Pericarpium Citri Reticulatae, and Pinellia ternata. Its main chemical constituents include succinic acid, hesperidin, ginsenoside Rb1, glycyrrhizic acid I, 2-atractylenolide, and pachymic acid. This decoction has been used to treat functional dyspepsia in traditional Eastern medicine. With its modulatory effects on gastrointestinal disturbance, this medicine is currently used clinically as a complementary therapy to attenuate cisplatin-induced side effects. Chiou et al. showed that this formula could attenuate CIPN symptoms in mice treated with platinum compounds through its anti-oxidative effect and regulatory action on mitochondria [119].

### 5.17. Other Herbal Formulas

Park et al. studied the nerve regeneration effect of Bogi Jetong Tang, a traditional decoction that consists of 18 medicinal ingredients, on a CIPN rat model [120]. Jeong et al. further showed the effects of Yideung Jetong Tang, which is a compressed version of Bogi Jetong Tang and consists of 12 ingredients, in a similar manner [121]. An et al. reported a case of a patient who experienced CIPN symptoms for over 2 years and was relieved by Ohjeok San treatment [122]. Hwanggi Gyeji Omul Tang (in Korean), also called Huang Qi Gui Zhi Wu Wu Tang (in Chinese) [123,124,125,200] and Ogi-keishi-gomotsu-To (in Japanese) [126], ameliorated symptoms of CIPN. Jakyak Gamcho Tang (in Korean), also called Shao Yao Gan Cao Tang (in Chinese) and Shakuyaku-kanzo-To (in Japanese), could help reduce CIPN symptoms [127,128,129].

## 6. Barriers in the Spread of Therapeutics for CIPN Based on Traditional Eastern Medicine

As introduced above, traditional medicinal approaches are frequently utilized to treat symptoms of CIPN in Asian countries and have shown significant results in clinical or preclinical settings. Nevertheless, therapeutic methods are not frequently introduced to the international community. The clinicians and patients in non-Asian countries have fewer opportunities to use those natural compounds that are under quality control by national healthcare systems, and the beneficial effects of the substances are sometimes controversial. As a result, these natural compounds ameliorating the adverse effects of chemotherapeutic agents tend to be used only in a subset of Asian countries. There exists several barriers that impede the spread of the traditional medicine against CIPN. Firstly, in the aspect of basic research, clinicians and researchers in non-Asian countries have less access to studies of traditional Eastern medicine. Studies on the traditional medicinal approach are fragmented according to the mixture of medicinal products (i.e., herbal formulas), natural materials that consist of the mixture, and individual compounds contained in the materials. In these circumstances, it is difficult for researchers and clinicians to know which substances should be of interest. Moreover, different Asian countries may have different names for each component. For example, research papers on herbal formulas use different notations depending on the country where the research was conducted (see alias of herbal formulas in Table 1). This makes it more difficult for Western researchers to search for and identify relevant studies. Next, from a clinical point of view, the issue of priority in the treatment arises. All CIPN patients have suffered from cancer, and the anti-cancer treatment inevitably becomes a priority for them. The patients and clinicians are often concerned about whether the interventions for CIPN may interfere with chemotherapy and/or facilitate cancer development. This makes them hesitant to try these seemingly speculative methods. They often decide to ignore CIPN symptoms or stick to the ineffective conventional treatments, rather than exploiting interventions in which the molecular pathways of action are not fully explained. To address this concern, future studies should provide detailed analgesic mechanisms along with more evidence that can prove the interventions do not exacerbate cancer. Clinical studies that can show strong statistical evidence are also needed.

## 7. Concluding Remarks

Knowledge of traditional Eastern medicine is useful not only for clinicians caring for patients with CIPN but also for researchers seeking new treatments. The substances derived from Eastern medicine can act on multiple targets with various mechanisms to exert analgesic effects against CIPN. In addition, some have anti-cancer effects on their own, and others can increase the efficiency of other co-treated anti-cancer drugs, with enhanced drug delivery and/or other molecular mechanisms. Generally, many substances act on multiple biological targets and exert beneficial effects, such as anti-inflammatory, anti-oxidative, anti-microbial, anti-obesity, neuroprotective, cardioprotective, and wound-healing effects. Utilizing this knowledge would help ameliorate the symptoms of patients and develop new treatments. However, we are not claiming that the treatments with natural compounds from traditional Eastern medicine substitute the chemotherapy. The current limitation is that the mechanisms involved in these actions have not been fully elucidated. More research is needed for a detailed understanding of the mechanisms underlying the molecular actions of the substances and for developing novel drugs based on this knowledge. The appropriate use of traditional knowledge would help in the discovery of efficient molecules in preclinical settings and lower the barrier to the development of new clinical pharmaceutics.

## Figures and Tables

**Table 1:** The substances of traditional Eastern medicine that have been exploited to treat CIPN. Per os (p.o.); subcutaneous (s.c.); intraperitoneal (i.p.); intrathecal (i.t.); visual analogue scale (VAS); numerical rating scale (NRS); Common Terminology Criteria for Adverse Events (CTCAE); National Cancer Institute-Common Toxicity Criteria for Adverse Events (NCI-CTCAE); Neurotoxicity Criteria of Debiopharm (DEB-NTC); chemotherapy-induced peripheral neuropathy assessment tool (CIPNAT).

Single Herbs and Phytochemicals			
Herb (Related Constituent)	Pain/CIPN Measures	Dose (Route of Administration)	Subject Chemotherapeutic Agent
Aconitum (Neoline, Bulleyaconitine A)	Mechanical, Cold	Processed aconite; 1 g/kg; 7 days; (p.o.) Water extract; 270 mg/kg; 7 days; (p.o.) Alkaloid fraction; 6 mg/kg; 7 days; (p.o.) Neoline 6 mg/kg; 7 days; (s.c.)	Mouse; Oxaliplatin [69]
		Processed aconite; 300 mg/kg; 5 days; (p.o.)	Rat; Oxaliplatin [65]
		Bulleyaconitine A; 0.1~0.8 mg/kg; 3 times a day; 7 days; (p.o.)	Rat; Paclitaxel [70]
		Processed aconite 30~100 mg/kg; (p.o.)	Rat Bortezomib [71]
Astragalus	Mechanical, Heat	300 mg/kg; (p.o.)	Rat; Oxaliplatin [72,73]
Coptis (Berberine)	Mechanical, Heat	Berberine 60~120 mg/kg; daily in the first 2 weeks and every other day after 2 weeks; 4 weeks observation; (p.o.)	Mouse; Cisplatin [74]
Cinnamomum (Coumarin, Cinnamic acid)	Mechanical, Cold	Cinnamomi cortex; 100~400 mg/kg; (p.o.) Coumarin; 10 mg/kg; (p.o.) Cinnamic acid; 10~40 mg/kg; (i.p.)	Rat; Oxaliplatin [66,67]
Curcuma (Curcumin)	Mechanical, Heat, Cold, Chemical (Formalin)	15~60 mg/kg; (p.o.)	Mouse; Vincristine [75]
		200 mg/kg; 5 weeks; (p.o.)	Rat; Cisplatin [76]
		12.5~50 mg/kg; 28 days; (p.o.)	Rat; Oxaliplatin [77]
Dryobalanops (Borneol)	Mechanical, Cold	15~60 μg/mouse; (i.t.)	Mouse; Oxaliplatin [78]
Lithospermum (Shikonin)	Mechanical	Lithospermi radix; 250 mg/kg; 4 weeks; (p.o.)	Mouse; Oxaliplatin [79]
Paeonia (Paeoniflorin)	Mechanical	0.1~1.0%; twice/day; 13 days; (transdermal)	Mouse; Paclitaxel [80]
Plantago (Aucubin, Pedicularis-lactone)	Mechanical	Plantaginis semen; 30~300 mg/kg; (p.o.) Aucubin; 15~100 mg/kg; (p.o.) Pedicularis-lactone; 15~100 mg/kg; (p.o.) Aucubin; 50 mg/kg; (i.p.)	Mouse; Paclitaxel [81,82,83,84]
Sophora (Matrine)	Mechanical, Pressure, Heat, Cold	Matrine; 15~60 mg/kg; 7~11 days; (i.p.)	Mouse; Vincristine [85,86]
**Bee Venom Therapy and Pharmacopuncture Therapies**			
**Substance** **(Related Constituent)**	**Pain/CIPN Measures**	**Dose** **(Route of Administration)**	**Subject** **Chemotherapeutic Agent**
Bee venom (Melittin, Phospholipase A2)	Mechanical, Cold	BV; 0.1~2.5 mg/kg; (ST36; s.c.) Phospholipase A2; 0.2 mg/kg; 5 days; (i.p.)	Mouse; Oxaliplatin [87,88,89]
		BV; 1.0~2.5 mg/kg + venlafaxine 40~60 mg/kg; (ST36; s.c.)	Mouse; Paclitaxel [90]
		BV; 1.0 mg/kg; (GV3, LI11, or ST36; s.c.) BV; 0.1 mg/kg; 18 days; (ST36; s.c.) BV; 0.25 mg/kg; (GV3; s.c.) Melittin; 0.5 mg/kg; (ST36; s.c.)	Rat; Oxaliplatin [91,92,93,94]
		BV; 1.0 mg/kg; (ST36; s.c.) Melittin; 0.5 mg/kg; (ST36; s.c.) Phospholipase A2; 0.12 mg/kg; (ST36; s.c.)	Rat; Paclitaxel [95]
		BV; 1.0 mg/kg; (ST36, s.c.)	Rat; Vincristine [96]
	Self report; VAS, Questionnaire, WHO CIPN grade	BV ointment; 1~2 times/day; (transdermal)	Human; Paclitaxel, Oxaliplatin, Carboplatin, Neoplatin [97]
		BV; 0.1 mL; 6 times; (GB39 and LV3 for lower extremities; LI4, SJ5, GB39, and LV3 for both upper and lower extremities; epidermal)	Human; Paclitaxel, Oxaliplatin, Cisplatin, Carboplatin [98]
		Melittin; 0.01 mg/acupoint; 3 times/week; (EX-UE9 and EX-LE10; epidermal)	Human; Paclitaxel, Carboplatin [99]
Snake venom	Self report; NRS, CTCAE	2.5 mg/acupoint; 4~8 times; (LI4 and TE3 for upper extremities; LR3 and GB41 for lower extremities; epidermal)	Human; Cisplatin [100]
Scolopendra subspinipes	Mechanical	0.5% solution; 20 ul; (ST36, s.c.)	Mouse; Oxaliplatin [101]
Toxicodendron vernicifluum	Self report; VAS, CIPNAT	1:1~3:2 mixture of dried resin of Toxicodendron vernicifluum (Rhus verniciflua stokes) and Cinnamomi cortex extracts; 0.2~0.5 mL/acupoint; 9 times; (multiple acupoints, epidermal)	Human; Cisplatin, Gemcitabine [102]
**Herbal Formulas**			
**Formulas** **(Alias)**	**Pain/CIPN Measures**	**Dose** **(Route of Administration)**	**Subject** **Chemotherapeutic Agent**
Gyeji ga Chul Bu Tang (Gui Zhi Jia Shu Fu Tang) (Keishi-ka-jutsu-bu-To)	Mechanical	200~600 mg/kg; 5 days; (p.o.)	Rat; Oxaliplatin [64]
	Self report; DEB-NTC	7.5 g/day + processed aconite 1~2 g; 2 weeks; (p.o.)	Human; Oxaliplatin [103]
Siwei Jianbu Tang	Mechanical, Heat, Cold	5~10 g/kg; preemptive; (p.o.)	Mouse; Oxaliplatin [104]
		5~10 g/kg; preemptive; (p.o.)	Mouse; Paclitaxel [105]
Ucha Shinki Hwan (Niu Che Shen Qi Wan) (Gosha-jinki-Gan) (Jeseng Singi Hwan) (Ji Sheng Shen Qi Wan)	Mechanical, Cold, Chemical (AITC, Menthol, Capsaicin)	0.3~1.0 g/kg; (p.o.)	Mouse; Oxaliplatin [106]
		0.1~1.0 g/kg; (p.o.)	Mouse; Paclitaxel [107]
		0.3~1.0 g/kg; +processed aconite 0.1~0.3 g/kg; (p.o.)	Rat; Oxaliplatin [108,109,110]
		450 mg/day; 21 days; (p.o.) 150 mg/kg; 5 weeks; preemptive; (p.o.)	Rat; Paclitaxel [111,112]
		0.3~1.0 g/kg; (p.o.)	Rat; Bortezomib [71]
	Electrical measure, Self report; VAS, NRS, Questionnaire, DEB-NTC, CTCAE, NCI-CTCAE	7.5 g/day; 14 days; (p.o.)	Human; Oxaliplatin [113,114,115,116,117]
		7.5 g/day; 6 weeks; (p.o.)	Human; Paclitaxel, Carboplatin [118]
Yukgunja Tang (Liu Jun Zi Tang) (Rikkunshi-To)	Mechanical	0.1~1.0 mg/kg; 6 days; preemptive; (p.o.)	Mouse; Paclitaxel
	Heat	0.1 mg/mL; 5 days/week; 3 weeks; preemptive; (p.o.)	Mouse; Cisplatin [119]
Bogi Jetong Tang	Nerve regeneration	400 mg/kg; 7 days; (p.o.)	Rat; Paclitaxel [120]
Yideung Jetong Tang	Nerve regeneration	400 mg/kg; 5 days; (p.o.)	Rat; Paclitaxel [121]
Ohjeok San	Self report; NRS, Questionnaire	Formula; 23.56 g; 3 times/day; 27 days; (p.o.)	Human; Bortezomib [122]
Hwanggi Gyeji Omul Tang (Huang Qi Gui Zhi Wu Wu Tang) (Ogi-keishi-gomotsu-To)	Mechanical, Heat, Cold	5.0~20.0 g/kg; 4 weeks; (p.o.)	Rat; Oxaliplatin [123]
	Self report; WHO CIPN grade, DEB-NTC, NCI-CTCAE	Modified formula; 3 times/week; 14 days; (External bath) Modified formula; twice/day; 21 days; (p.o.) Dose unknown; 4 weeks; (p.o.)	Human; Oxaliplatin [124,125,126]
Jakyak Gamcho Tang (Shao Yao Gan Cao Tang) (Shakuyaku-kanzo-To)	Mechanical	1.75 mg/day; 5 days; preemptive; (p.o.)	Mouse; Paclitaxel [127]
	Self report	7.5 g/day; 7~8 days; preemptive; (p.o.)	Human; Paclitaxel, Carboplatin [128,129]
